# Supplementary material for: Increase in stable isotope ratios driven by metabolic alterations in amphipods exposed to the beta-blocker propranolol
Source: PLoS One. 2019 May 16;14(5):e0211304. doi: 10.1371/journal.pone.0211304 (PMC6522046; doi:10.1371/journal.pone.0211304)
Supplement: S1 File — Additional information on the observed mortality and propranolol levels in amphipod and water samples (Table A), statistical evaluation of the aquarium effects on the measured endpoints (Table B), a summary of the measured endpoints (Table C) and a diagram illustrating pathways leading to isotopic fractionation and affected by propranolol (Figure A). (PDF) [file pone.0211304.s001.pdf]

Supporting Information, S1 File

Increase in stable isotope ratios driven by metabolic alterations in amphipods exposed to the beta-blocker propranolol

Caroline Ek[a,], Zhenyang Yu[b], Andrius Garbaras[c], Hanna Oskarsson[d], Ann-Kristin Eriksson Wiklund[a], Linda Kumblad[d] and Elena Gorokhova[a]*

[a]Department of Environmental Science and Analytical Chemistry, Stockholm University, Svante Arrhenius väg 8, SE-106 91 Stockholm, Sweden

[b]State Key Laboratory of Pollution Control and Resource Reuse, Key Laboratory of Yangtze River Water Environment, Ministry of Education, College of Environmental Science and Engineering, Tongji University, 200092 Shanghai, P. R. China

[c]Mass Spectrometry Laboratory, Center for Physical Science and Technology, Savanoriu 231, LT-02300 Vilnius, Lithuania

[d]Department of Ecology, Environment and Plant Science, Stockholm University, Svante Arrhenius väg 20, SE-114 18 Stockholm, Sweden

*Corresponding author:

Elena Gorokhova

[a]Department of Environmental Science and Analytical Chemistry, Stockholm University, Svante Arrhenius väg 8, SE-106 91 Stockholm, Sweden

Table A. Mortality (% dead) and concentrations of propranolol quantified in amphipods (μg g WW^{-1}) and water (μg L^{-1}) in experimental treatments; see Oskarsson et al. [1] for details on methods and samples. Values are expressed as mean±SE. Treatments: control (0 μg L^{-1} propranolol), PL (100 μg L^{-1} propranolol), PH (1000 μg L^{-1} propranolol). N.a., not available.

Treatment	Mortality	Water	Amphipods
Control	77±6.1	<LOQ	<LOQ
PL	64±12	108±5.8	3.2±n.a.
PH	51±9.6	1058±37	6.3±n.a.

Table B. Model comparisons using ANOVA to elucidate aquarium effect (as a random factor) of propranolol exposure on the response variables %C, %N, C:N ratio, protein, WW, TBARS, ORAC, ORAC:TBARS ratio, AChE, $\delta^{15}N$ and $\delta^{13}C$. Model 1: linear mixed model with aquarium effect; Model 2: generalised least square without aquarium effect. L ratio: likelihood ratio. Significant aquarium effects are shown in bold.

Variable	Model	df	AIC	L ratio	p-value
%C	1	4	203.4324		
	2	3	206.1138	4.6814	**0.0305**
%N	1	4	100.9439		
	2	3	98.95248	0.0086	0.9260
C:N	1	4	29.17546		
	2	3	31.07789	3.9024	**0.0482**
Protein	1	4	43.18696		
	2	3	41.18696	0.0000	0.9999
WW	1	4	-240.758		
	2	3	-242.701	0.0570	0.8112
TBARS	1	4	4.87399		
	2	3	3.100907	0.2269	0.6338
ORAC	1	4	-95.4723		
	2	3	-97.4723	0.0000	0.9999
ORAC:TBARS	1	4	67.0875		
	2	3	65.0875	0.0000	0.9999
AChE	1	4	-853.14		
	2	3	-855.14	0.0000	0.9997
$\delta^{15}N$	1	4	72.79188		
	2	3	70.79188	0.0000	0.9999
$\delta^{13}C$	1	4	120.6878		
	2	3	118.6878	0.0000	0.9999

Table C. Data summary for biochemical variables and stable isotopes measured in the propranolol-exposed amphipods and in the controls; values are expressed as mean ± SE. Treatments, control (0 μg L^{-1} propranolol), PL (100 μg L^{-1} propranolol), PH (1000 μg L^{-1} propranolol); Mortality, expressed in % after 6 weeks of the experiment; %N, nitrogen content; C:N, ratio between %C and %N; Protein content (mg mL^{-1}); WW, wet weight (mg); TBARS, thiobarbituric acid reactive substances (μM MDA mg $protein^{-1}$); ORAC, oxygen radical absorbance capacity (mg trolox eq. mg $protein^{-1}$); ORAC:TBARS, balance between antioxidative and pro-oxidative activities, AChE, acetylcholinesterase activity (nmol AsSCh mg $protein^{-1}$ min^{-1}). Values of $\delta^{15}N$ and $\delta^{13}C$ are expressed in ‰. *: Modified from Oskarsson and co-workers [1].

		Mortality	%N	%C	C:N	Protein	WW	TBARS	ORAC	ORAC:TBARS	AChE	$\delta^{15}N$	$\delta^{13}C$
Control													
	mean	77	8.23	35.39	4.31	1.20	41.6	0.47	0.29	0.81	34.5	4.1	-23.6
	SE	6.1	0.13	0.51	0.08	0.09	3.4	0.06	0.02	0.12	3.5	0.07	0.2
PL													
	mean	64	8.18	34.70	4.25	1.17	36.7	0.39	0.29	0.84	29.8	4.2	-23.3
	SE	12	0.15	0.48	0.04	0.05	1.7	0.03	0.01	0.09	3.1	0.1	0.1
PH													
	mean	51	8.58	35.97	4.21	1.05	35.2	0.33	0.26	0.97	30.3	4.8	-22.6
	SE	9.6	0.16	0.53	0.07	0.08	2.7	0.05	0.01	0.09	3.7	0.1	0.2

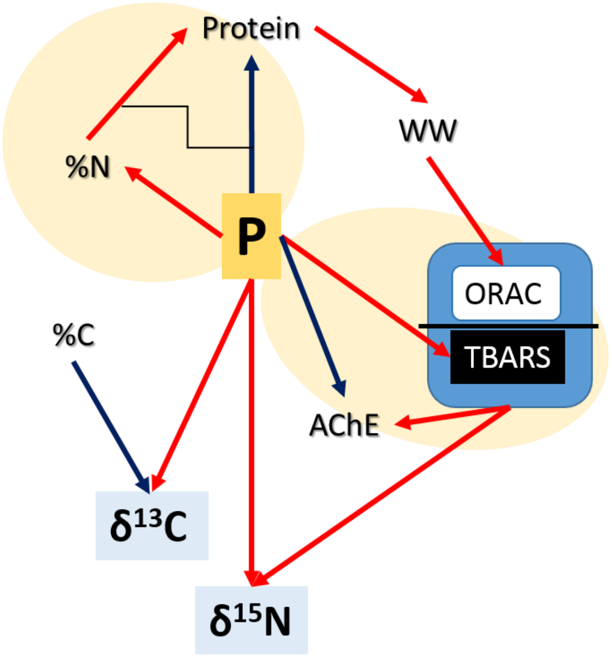

Figure A. Significant effects on amphipod growth and its constituents, biomarkers and stable isotope signatures, induced by propranolol (P). The effects are shown as arrows pointing from a predictor to the dependent variable. Positive effects are shown as red arrows, negative – as black arrows. Interaction between the predictors is shown as a thin broken line. The yellow fields indicate effects predicted on the basis of MOA. Protein, percentage of protein in the body; WW, wet weight; %N and %C, percentage of nitrogen and carbon, respectively; AChE, acetylcholinesterase activity; ORAC, antioxidant capacity; TBARS, lipid peroxidation; ORAC:TBARS ratio, oxidative balance (shown as a blue box); $\delta^{13}C$ and $\delta^{15}N$, stable isotope signatures of the amphipods. See Table 1 for the rationale for specific effects.

Reference

1. Oskarsson H, Wiklund A-KE, Thorsén G, Danielsson G, Kumblad L. Community Interactions Modify the Effects of Pharmaceutical Exposure: A Microcosm Study on Responses to Propranolol in Baltic Sea Coastal Organisms. PLOS ONE. 2014;9: e93774. doi:10.1371/journal.pone.0093774
